# Genome-Wide Identification, Expression, and Functional Analysis of the Sugar Transporter Gene Family in Cassava (*Manihot esculenta*)

**DOI:** 10.3390/ijms19040987

**Published:** 2018-03-26

**Authors:** Qin Liu, Huijie Dang, Zhijian Chen, Junzheng Wu, Yinhua Chen, Songbi Chen, Lijuan Luo

**Affiliations:** 1 Hainan Key Laboratory for Sustainable Utilization of Tropical Bioresources, Institute of Tropical Agriculture and Forestry, Hainan University, Haikou 570110, China; 11071001110002@hainu.edu.cn (Q.L.); dhj1990@hainu.edu.cn (H.D.); tang_zhijuan@hainu.edu.cn (J.W.); yhchen@hainu.edu.cn (Y.C.); 2 Institute of Tropical Crop Genetic Resources, Chinese Academy of Tropical Agriculture Sciences, Danzhou 571737, China; zjchen@catas.cn (Z.C.); songbichen@catas.cn (S.C.)

**Keywords:** cassava, sugar transporter, gene family, sugar unloading, tuber root

## Abstract

The sugar transporter (*STP*) gene family encodes monosaccharide transporters that contain 12 transmembrane domains and belong to the major facilitator superfamily. *STP* genes play critical roles in monosaccharide distribution and participate in diverse plant metabolic processes. To investigate the potential roles of *STPs* in cassava (*Manihot esculenta*) tuber root growth, genome-wide identification and expression and functional analyses of the *STP* gene family were performed in this study. A total of 20 *MeSTP* genes (*MeSTP1*–*20*) containing the Sugar_tr conserved motifs were identified from the cassava genome, which could be further classified into four distinct groups in the phylogenetic tree. The expression profiles of the *MeSTP* genes explored using RNA-seq data showed that most of the *MeSTP* genes exhibited tissue-specific expression, and 15 out of 20 *MeSTP* genes were mainly expressed in the early storage root of cassava. qRT-PCR analysis further confirmed that most of the *MeSTPs* displayed higher expression in roots after 30 and 40 days of growth, suggesting that these genes may be involved in the early growth of tuber roots. Although all the MeSTP proteins exhibited plasma membrane localization, variations in monosaccharide transport activity were found through a complementation analysis in a yeast (*Saccharomyces cerevisiae*) mutant, defective in monosaccharide uptake. Among them, MeSTP2, MeSTP15, and MeSTP19 were able to efficiently complement the uptake of five monosaccharides in the yeast mutant, while MeSTP3 and MeSTP16 only grew on medium containing galactose, suggesting that these two MeSTP proteins are transporters specific for galactose. This study provides significant insights into the potential functions of *MeSTPs* in early tuber root growth, which possibly involves the regulation of monosaccharide distribution.

## 1. Introduction

Sugar, regarded as an essential substrate in carbon and energy metabolism, plays diverse roles in plant growth and development. Sugars also act as signal molecules for signal transduction and as important elements for the synthesis of cellular compounds, contributing to the regulation of osmotic homeostasis [1]. In plants, sugars are transported from source tissues (e.g., leaves) to sink tissues (such as roots, seeds, and other reproductive tissues) mainly in the form of sucrose, polyols, or oligosaccharides. As the major carbohydrate in plants, sucrose is mainly synthesized in the mesophyll. After allocation to the collection phloem, sucrose is translocated via a long-distance transport system in the transport phloem and finally transported to the release phloem. A variety of strategies have been evolved by plants for the release of sucrose from the phloem to the sink tissues. For example, at the release phloem, sucrose can be transported to sink cells via a symplastic pathway through plasmodesmata or via an apoplastic pathway mediated by sucrose transporters, cell wall invertases, and monosaccharide transporters [2]. The uptake of hexose (e.g., glucose and fructose) hydrolyzed from sucrose in the apoplast is regulated by a group of transporters, such as sugar transporter proteins (STPs) in Arabidopsis, monosaccharide transporters (MSTs) in rice (*Oryza sativa*), and hexose transporters (HTs) in grape (*Vitis vinifera*), which are involved in sugar unloading and thus contribute to carbon partitioning, crop yield, and environmental adaptation [3].

STP proteins belong to the major facilitator superfamily commonly possessing 12 transmembrane domains and are regarded as H^+^/sugar symporters [4]. Since the first *STP* gene was cloned from *Chlorella* [5], a set of *STP* genes have been identified and characterized from other plant species, such as Arabidopsis, rice, grape, tomato (*Lycopersicon esculentum*), and pear (*Pyrus bretschneideri*) [6,7,8,9,10,11]. Most of the characterized STP proteins are plasma membrane-localized transporters and exhibit acquisition of broad monosaccharide substrates, such as glucose, fructose, galactose, xylose, and mannose [6]. Cumulative results demonstrate that *STP* genes display different expression patterns during plant growth and respond to stress conditions. In Arabidopsis, for example, *AtSTP1* is mainly expressed in germinating seeds, young seedlings, and guard cells [12]. *AtSTP2*, *AtSTP6*, *AtSTP9*, and *AtSTP11* show tissue-specific expression in pollen, while *AtSTP4* exhibits the highest expression in roots [6,13]. *AtSTP13*, localized to the vasculature and leaf mesophyll cells, probably participates in the retrieval of sugars leaked from the cytoplasm [14]. In rice, *OsMST3* is expressed specifically in the root xylem involved in the accumulation of monosaccharides required for cell wall synthesis at the stage of cell thickening [7]. Furthermore, *STP* genes also respond to environmental stresses, such as wounding [13,15], nematode infection [16], and pathogen attack [17]. Although the expression patterns and functional analysis of *STP* genes have been previously studied, the comprehensive expression profiles of the *STP* gene family in root crops, such as cassava (*Manihot esculenta*), are poorly characterized and may provide significant insights into the various roles of *STPs* in different plant species.

Cassava is the third largest source of food cultivated in tropical and subtropical areas, providing food for more than 800 million people globally [18]. As its high starch content accumulates in tuber roots, cassava is a major source for the industrial production of starch and bioethanol [19,20]. In the past two decades, numerous in silico data were generated and used for the screening of potential genes of particular traits in cassava, including those involved in cyanogenic glucoside synthesis [21], post-harvest physiological deterioration [22], storage roots development [23], biotic and abiotic stress responses [24,25]. All those data, along with the cassava genome, guarantee the possibility to analyze gene structure, chromosome location, phylogeny, evolutionary patterns, and expression profiles of gene families at a genome-wide level, as exemplified by the studies of *MeFRK*, *MeHXK*, *MeWRKY*, and *MeMAPK* families [26,27,28,29]. However, little information on *STP* gene family expression in different tissues and developmental roots of cassava is available. Because of the vital functions of *STPs* in plants, it is of considerable importance to investigate the *STP* gene family in cassava. In this study, the *MeSTP* gene family was identified from cassava genome, and a in silico analysis of *MeSTP* genes was conducted in detail. The expression profiles of *MeSTP* genes in different tissues and in the developmental roots of cassava were explored using the publicly available transcriptome data. The expression of *STP* genes in the roots was then confirmed by quantitative RT-PCR (qRT-PCR) analysis. Furthermore, functional and subcellular localization analyses of MeSTPs were performed. 

## 2. Results

### 2.1. Identification of the STP Gene Family in Cassava

In this study, a total of 20 putative *MeSTP* genes were identified in the cassava genome through a BLAST search and HMMER analysis. General information for the 20 *MeSTP* members is summarized in Table 1. The 20 *MeSTP* genes were unevenly located on cassava chromosomes and thus they were named *MeSTP*1 to *MeSTP*20, according to their chromosomal positions (Table 1). Among them, chromosome 1, 3, and 15 had three *MeSTPs* each, chromosomes 2, 6, and 16 contained two *MeSTPs* each, and chromosomes 4, 11, 14, 17, and 18 contained one *MeSTP* each (Table 1). The open reading frames (ORFs) of the *MeSTP* genes ranged from 1326 to 1593 bp in length (Table 1). The length of the MeSTP proteins ranged from 442 to 531 amino acids, with predicted molecular weights from 48.74 kDa to 58.54 kDa. The theoretical isoelectric point (p*I*) of the MeSTP proteins ranged from 6.84 to 10.06. Furthermore, 19 of the 20 MeSTPs harbored 10 to 12 conserved transmembrane domains (TMDs), while MeSTP18 carried only 8 TMDs (Table 1).

### 2.2. Sequence Structure Features of MeSTPs

**Figure 1:**
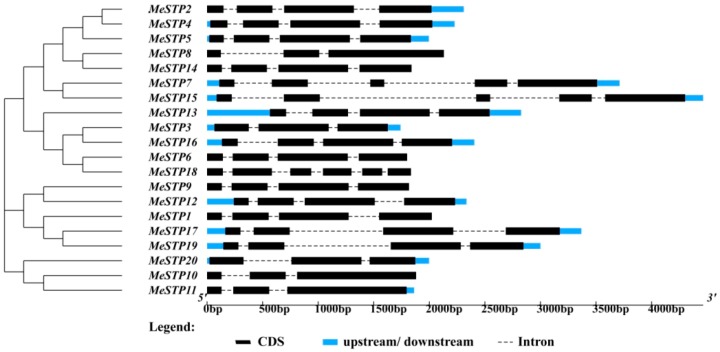
Exon–intron structures of the 20 *MeSTP* genes generated from the GSDS website (http://gsds.cbi.pku.edu.cn/chinese.php). The neighbor-joining phylogenetic tree of the *MeSTPs* gene sequences was constructed using 1000 bootstrap replicates by MEGA v7.0 (Pennsylvania State University, Philadelphia, PA, USA).

The results showed that the 20 *MeSTP* genes could be classified into three distinct groups according to the similarity of their gene sequences (Figure 1). The largest group contained 12 *MeSTP* genes, while the other two groups contained 5 and 3 *MeSTP* genes, respectively (Figure 1). The structure of the *MeSTP* genes commonly presented four exons divided by three introns, except for *MeSTP*7, *MeSTP*8, *MeSTP*10, *MeSTP*11, *MeSTP*15, and *MeSTP*20 (Figure 1). Of these, *MeSTP8* together with *MeSTP*10, *MeSTP*11, and *MeSTP*20 possessed three exons and two introns. *MeSTP*7 and *MeSTP*15 contained five exons divided by four introns, while *MeSTP*18 had six exons and five introns (Figure 1). Furthermore, most of the *MeSTP* genes classified into the same subgroup exhibited similar gene structures, except for *MeSTP6* and *MeSTP*18, which possessed four exons and six exons, respectively (Figure 1).

**Figure 2:**
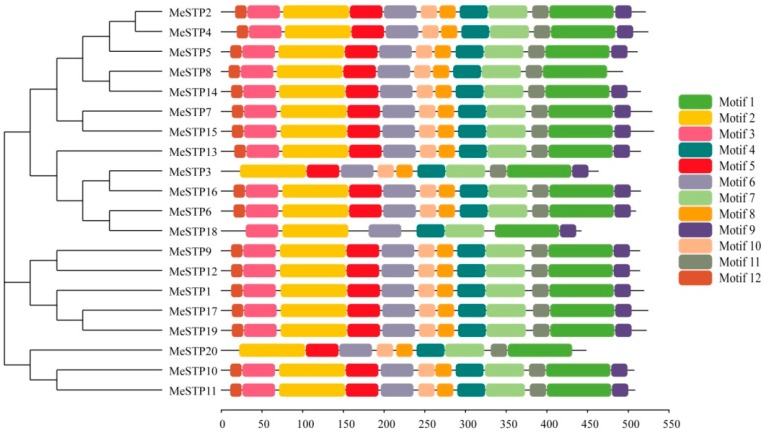
Conserved motifs of the MeSTP proteins identified by MEME. The gray lines represent the non-conserved sequences, and each motif is indicated by a colored box numbered at the bottom. The lengths of the motifs in each protein are proportional. The neighbor-joining phylogenetic tree of MeSTPs protein sequences was constructed using 1000 bootstrap replicates by MEGA 7.0.

Conserved motifs of the MeSTP proteins were identified using the MEME program, and the motif sequences and annotations were further predicted by Pfam. The results showed that 12 putative conserved motifs were identified in most of the MeSTP proteins (Table 2 and Figure 2). The length of the conserved motifs ranged from 15 to 82 amino acids, and putative Sugar_tr domains were predicted in conserved motifs 1 through 7 of the MeSTP proteins (Table 2). Furthermore, motifs 1, 2, 3, 4, 6, and 7, harboring Sugar_tr domains, exhibited high conservation in the twenty MeSTP proteins (Figure 2). It was found that all of the MeSTP proteins contain the 12 motifs, except for MeSTP3, MeSTP18, and MeSTP20. For example, MeSTP3 misses motif 12 in the N-terminal region, and MeSTP20 lacks motif 9 in the C-terminal region. MeSTP18 lacks motifs 5, 8, 10, 11, and 12 in the middle of the protein sequence (Figure 2).

### 2.3. Phylogenetic Analysis of MeSTP Proteins

**Figure 3:**
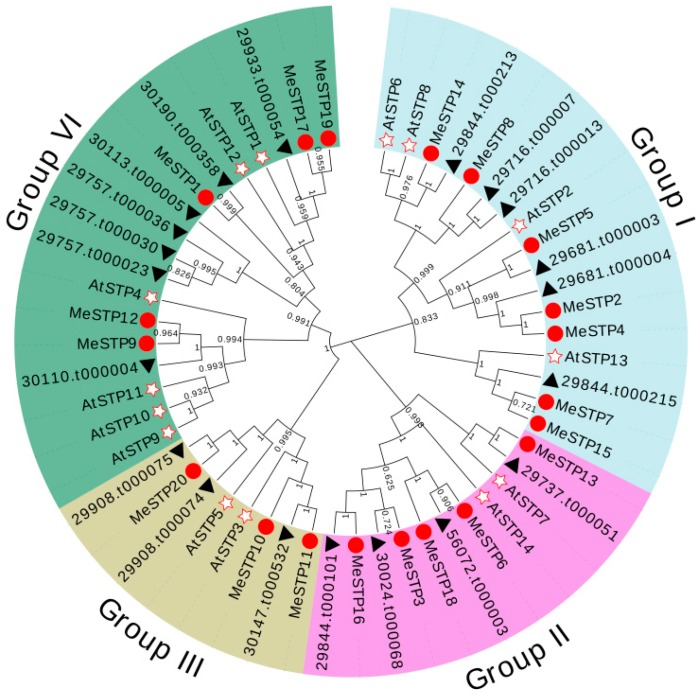
Phylogenetic relationship of STP proteins in three plant species. The evolutionary relationship is presented using a phylogenetic tree. STP proteins (MeSTP1-MeSTP20) from cassava, *Arabidopsis thaliana* (AtSTP1-AtSTP14), and *Ricinus communis* are marked with red circles, hollow stars, and black triangles, respectively. The accession numbers are listed in Supplementary Table S2.

A phylogenetic tree was constructed to analyze the evolutionary relationships among STP proteins from cassava, Arabidopsis, and castor bean. The results showed that the STP proteins could be classified into four groups, and MeSTPs grouped with AtSTPs and RcSTPs in each group (Figure 3). Among them, seven MeSTPs (MeSTP2, 4, 5, 7, 8, 14 and 15) together with four AtSTPs from Arabidopsis and six RcSTPs from castor bean were attributed to Group I (Figure 3). Group II consisted of five MeSTPs (MeSTP3, 6, 13, 16 and 18), two AtSTPs, and four RcSTPs (Figure 3). Three MeSTPs (MeSTP10, 11, and 20) clustered with two AtSTPs and three RcSTPs in Group III (Figure 3). Group IV contained five MeSTPs (MeSTP1, 9, 12, 17, and 19), six AtSTPs, and seven RcSTPs (Figure 3). Furthermore, a total of five sister pairs of MeSTPs were observed in the phylogenetic tree, including MeSTP2–MeSTP4, MeSTP6–MeSTP18, MeSTP7–MeSTP15, MeSTP9–MeSTP12, and MeSTP17–MeSTP19 (Figure 3).

### 2.4. Gene Duplication Analysis

**Figure 4:**
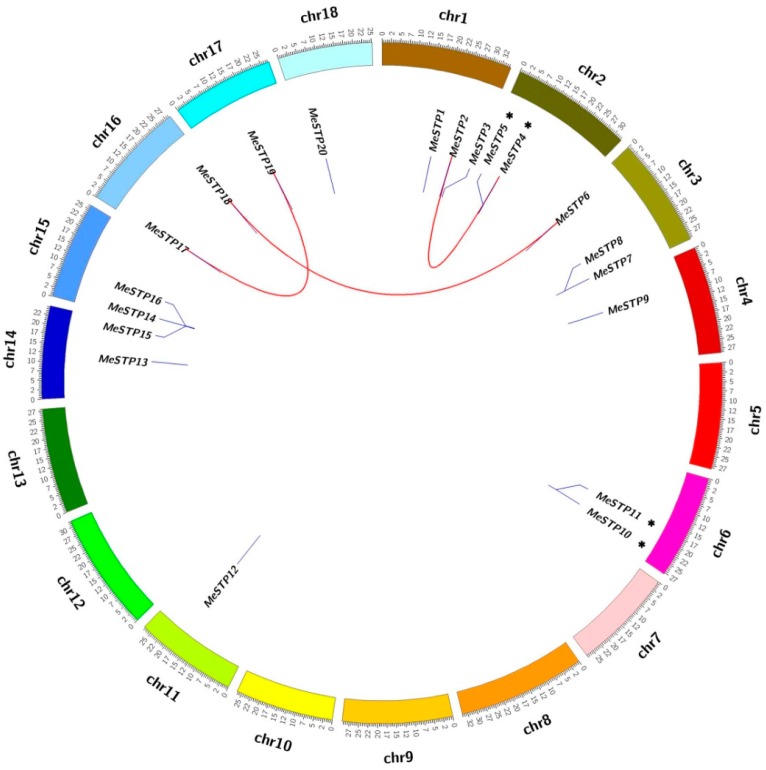
Chromosomal distribution and gene duplications of the *MeSTP* genes. The scale on the circle is in Megabases. Each colored bar represents a chromosome as indicated. Gene IDs are labeled on the basis of their positions on the chromosomes. The putative whole genome duplication (WGD) or segmental duplication genes are linked by a red line. Asterisks indicate tandem duplication.

Subsequently, the tandem duplications and whole genome duplications (WGDs/segmental) of the *MeSTP* genes were analyzed. As shown in Figure 4, the *MeSTP* genes were differentially distributed on 11 out of 18 cassava chromosomes. Among these *MeSTP* genes, three pairs of genes, including *MeSTP*2*–MeSTP*4, *MeSTP*6*–MeSTP*18, and *MeSTP*17*–MeSTP*19, exhibited WGD/segmental duplication. Two pairs of genes, *MeSTP*4*–MeSTP*5 and *MeSTP*10*–MeSTP*11, were regarded as tandem duplication genes (Figure 4).

### 2.5. Expression Profiles of MeSTP Genes

**Figure 5:**
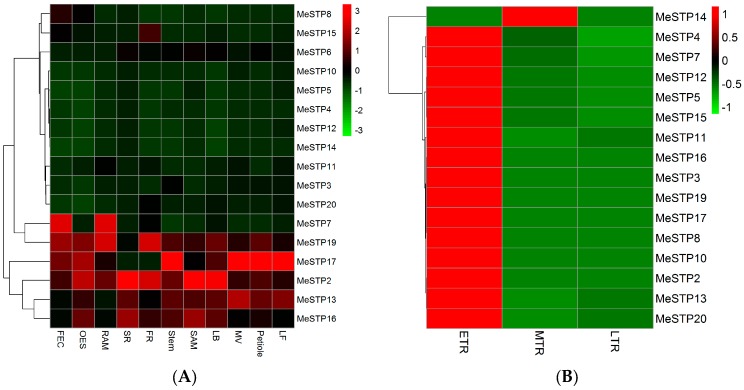
The expression profiles of *MeSTP* genes. The heat map of the *MeSTP* gene expression levels was hierarchically clustered using the R package “pheatmap” with the data normalization method of Z-score standardization. The color scale bar, ranging from green to red, represents low and high expression, respectively. (**A**) Expression profiles of *MeSTP* genes in 11 tissues, including friable embryogenic callus (FEC), somatic organized embryogenic structures (OES), root apical meristem (RAM), storage root (SR), fibrous root (FR), stem, shoot apical meristem (SAM), lateral bud (LB), midvein (MV), petiole, and leaf (LF); (**B**) expression profiles of *MeSTP* genes at the three different growth stages of roots, including the early storage root (ETR), the medium tuber root (MTR), and the late storage root (LTR).

The cassava RNA-seq data from the GEO database, which were submitted by Wilson et al. [30], were used to explore the expression profiles of *MeSTP* genes in eleven tissues. As three *MeSTPs* (*MeSTP1*, *MeSTP*9, and *MeSTP*18) genes were not expressed in any tissues of cassava, the expression of the remaining 17 *MeSTPs* was considered for hierarchical clustering analysis. The results showed that *MeSTP* genes exhibited differential expression in various cassava tissues (Figure 5A). Among them, *MeSTP2* and *MeSTP*20 exhibited tissue-specific expression in the sink tissue (fibrous root, FR), while *MeSTP5* and *MeSTP8* showed the highest expression in leaf (LF) and friable embryogenic callus (FEC), respectively (Figure 5A). Furthermore, six *MeSTPs* (*MeSTP*2, *MeSTP*7, *MeSTP*13, *MeSTP*15, *MeSTP*17, and *MeSTP*19) exhibited higher expression levels in a specific tissue compared to the remaining *MeSTPs*. For example, *MeSTP*2 showed a higher expression in the sink tissues (e.g., FR, lateral bud (LB), organized embryogenic structures (OES), shoot apical meristem (SAM), and storage root (SR)), while *MeSTP*17 displayed a higher expression in LF, midvein (MV), petiole, and stem, compared to the other *MeSTP* genes (Figure 5A).

Cassava RNA-seq data from the public SRA database submitted by the Chinese Academy of Tropical Agricultural Sciences [21] were used to dissect the expression profiles of *MeSTPs* in the roots at three growth stages. As four *MeSTPs* (*MeSTP1*, *MeSTP6*, *MeSTP9,* and *MeSTP*18) were not expressed in the roots at any growth stages, a total of 16 *MeSTP* genes were used for further analysis (Figure 5B). The results showed that 15 out of 16 *MeSTP* genes were mainly expressed in the early storage roots, followed by the medium tuber root, and the late storage roots (Figure 5B). For example, the expression level of *MeSTP2* in the early storage roots was 11-fold and 10-fold higher than that in the medium tuber roots and the late storage roots, respectively (Figure 5B).

**Figure 6:**
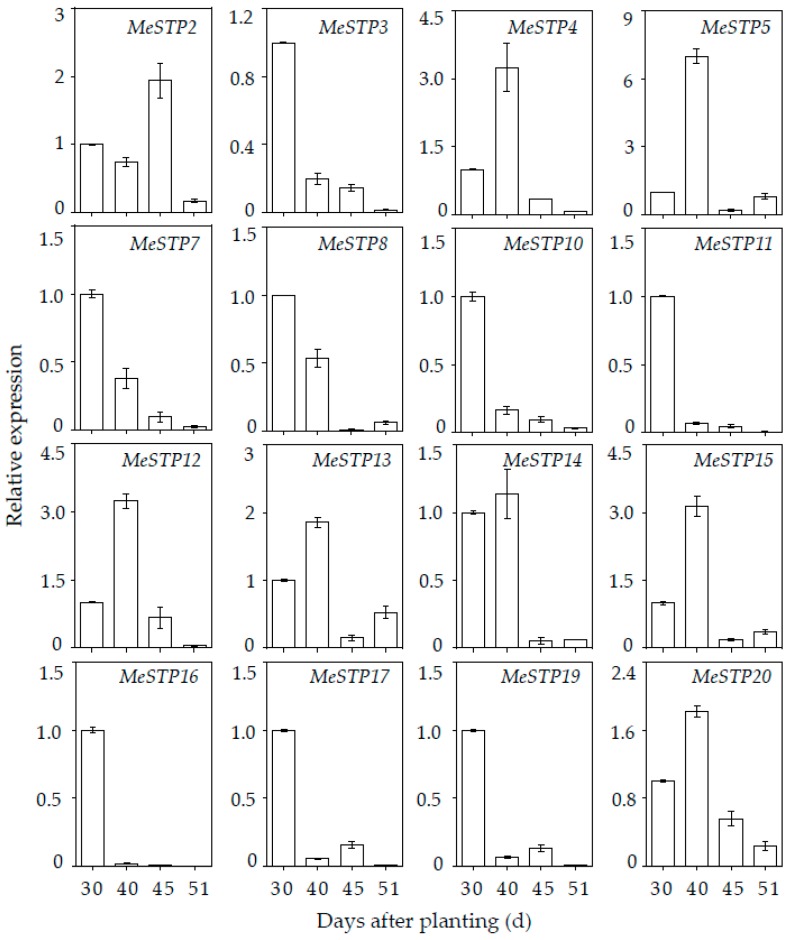
Expression patterns of sixteen *MeSTP* genes in the roots. qRT-PCR was conducted to analyze *MeSTP* gene expression in the roots at 30, 40, 45, or 51 days of growth in normal conditions. The gene expression in the roots at 30 days of growth was set to one, and the expression level of each gene was normalized with respect to its expression in the roots at 30 days of growth.

Subsequently, a qRT-PCR analysis was performed to verify the expression of 16 *MeSTPs*, as outlined in the previous paragraph, in the roots at 30, 40, 45, and 51 days of growth. The results showed that most of the *MeSTPs* displayed higher expression in the roots at 30 and 40 days of growth (Figure 6). For example, eight *MeSTPs* and seven *MeSTPs* showed higher expression in the roots at 30 and 40 days of growth, respectively, compared to their expression in the roots at a later stage (Figure 6). Furthermore, all of the tested *MeSTPs* exhibited decreased expression in the roots after 45 days of growth, except for *MeSTP2*, which displayed the highest expression in the roots at 45 days of growth, followed by a decreased expression at 51 days of growth (Figure 6). These results suggest that some of the *MeSTP* genes may be involved in the early root growth.

### 2.6. Complementation Analysis of MeSTPs in a Yeast Strain Defective in Monosaccharide Uptake

**Figure 7:**
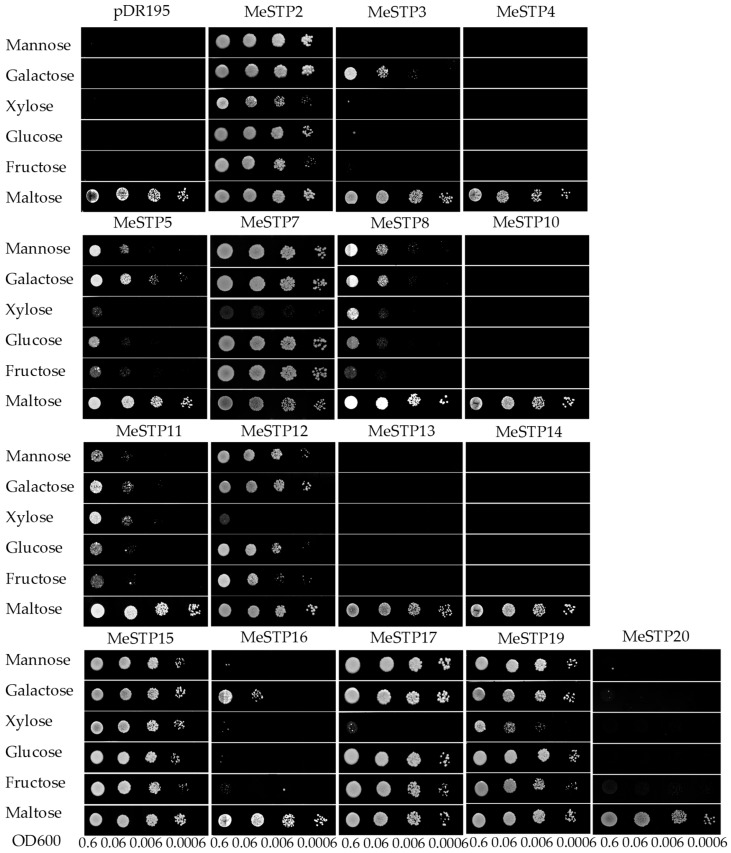
Complementation of yeast EBY. VW4000 mutant by *MeSTP* genes. Yeast cell suspensions with optical density (OD) at 600 nm of 0.6 and three corresponding 1:10 dilutions were dropped on solid SD-ura medium containing 2% mannose, galactose, xylose, glucose, or fructose for 3 days at 27 °C; pDR195 indicates yeast cells transformed with the empty vector pDR195.

To further analyze and compare the monosaccharide transport activity of *MeSTPs*, 16 out of 20 *MeSTPs* were successfully cloned and separately introduced into yeast strain EBY.VW4000, which is deficient in monosaccharide uptake ability. Five monosaccharide substrates, including glucose, fructose, mannose, galactose, and xylose, were used to examine the sugar transport activity of *MeSTPs*. As shown in Figure 7, all yeast cells grew well on synthetic deficient (SD) media containing 2% maltose, suggesting the presence of the expression vectors containing the target genes. The control yeast cells transformed with empty vector pDR195 could not grow on SD medium containing any of the tested monosaccharides (Figure 7). Among the yeast cells carrying *MeSTPs*, three *MeSTP* members (*MeSTP*2, 15, and 19) could efficiently complement the yeast mutant in the SD medium containing the five tested monosaccharides, while *MeSTP*5, *MeSTP*8, and *MeSTP*11 transformants grew weakly but much better than the pDR195 control when supplied with these monosaccharide substrates. Three *MeSTP* (*MeSTP*7, 12, and 17) transformants grew well on SD medium supplemented with mannose, galactose, glucose, and fructose, but not xylose. However, yeast cells carrying *MeSTP*3 and *MeSTP*16 only grew on SD medium containing galactose, suggesting that these two *MeSTP* proteins function as galactose-specific transporters in yeast. Furthermore, the remaining five *MeSTP* members (*MeSTP*4, 10, 13, 14, and 20) failed to induce yeast growth on SD medium containing the five tested monosaccharides (Figure 7), suggesting that these five *MeSTP* members could not transport these substrates in yeast.

### 2.7. Subcellular Localization of MeSTPs

**Figure 8:**
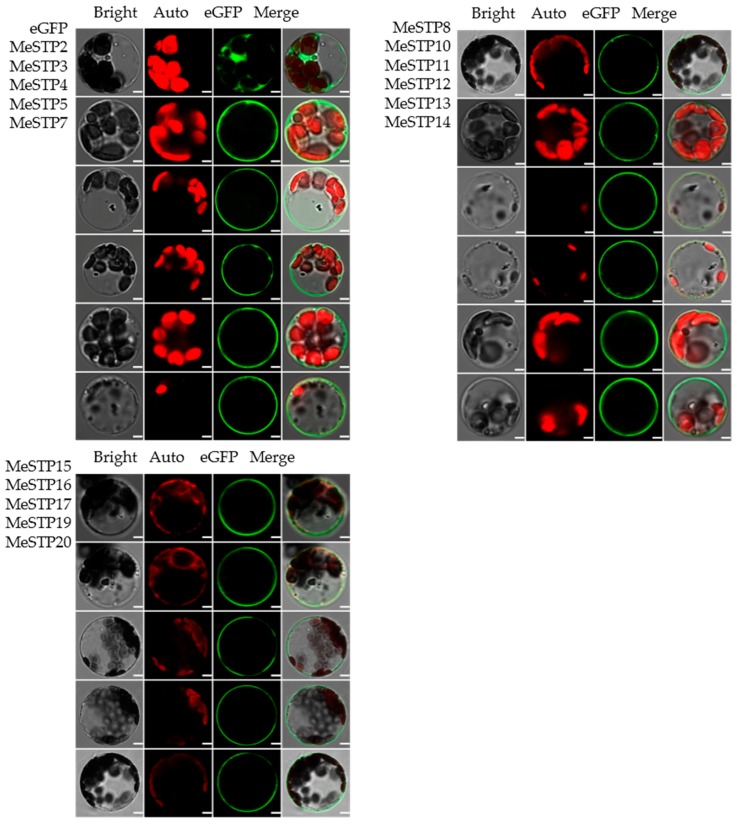
Subcelluar localization of MeSTPs proteins in cassava mesophyll protoplasts. The cassava mesophyll protoplasts expressing the empty vector pX-DG were used as a control. Bright-field images (Bright), chlorophyll autofluorescence (Auto), fluorescence of eGFP fusion protein (eGFP), and te merged images (Merge) were examined using laser-scanning confocal microscopy. Bars = 5 μm.

To analyze the subcellular localization of MeSTP proteins, MeSTP-GFP fusion proteins were transiently expressed in cassava mesophyll protoplasts. The results showed that the signal of GFP in cells transfected with the empty vector was mainly detected in the cytoplasm and plasma membrane, whereas the GFP signals in cells expressing the 16 MeSTP fusion proteins were confined to the plasma membrane (Figure 8), suggesting that these MeSTPs might be membrane proteins.

## 3. Discussion

STP proteins in model plants, such as Arabidopsis and rice, have been shown to play critical roles in sugar transport [6,31]. However, the genome-wide expression profile of the *STP* gene family is less well defined in other crops, such as cassava, which is characterized by a high starch content in the roots. The release of a high-quality cassava genome provides an opportunity to explore the *MeSTP* gene family and to dissect its potential roles in root growth. In this study, gene structure, gene duplications, and protein motifs of the MeSTP family were globally analyzed in cassava for the first time. Furthermore, the expression pattern and function analysis of the *MeSTP* genes were further investigated.

A total of 20 *MeSTP* genes were identified in the cassava genome through a BLAST search and HMMER analysis (Table 1). Most of the cassava STP proteins harbored a Sugar_tr domain (PF00083) belonging to the major facilitator superfamily (MFS) (Table 2). MFS transporters in plants possess a common structure of 12 transmembrane domains (TMD1–TMD12), which are separately contained within the N-domain (TMD1–TMD6) and the C-domain (TMD7–TMD12) [4,32]. In the current study, 10 out of 20 MeSTP proteins contained the entire 12 TMDs, while 8 MeSTP members possessed 10 or 11 TMDs. However, MeSTP18 carried only eight TMDs, displaying a sequence deletion in the middle of the protein (Table 1). These results suggest that loss of the N-terminal or C-terminal regions may have occurred in some MeSTP members during evolution. Consistent with this, similar STP protein structures are also observed in tomato and grape [9,10]. Furthermore, most of the *MeSTP* genes contained four exons and three introns, except for seven *MeSTPs* (*MeSTP*7, *MeSTP*8, *MeSTP*10, *MeSTP*11, *MeSTP*15, *MeSTP*18, and *MeSTP*20), which displayed variations in the numbers of exons and introns (Figure 1). Similarly, the numbers of exons and introns in *STP* genes have been demonstrated to range from three to five in other plant species, such as Arabidopsis, grape, and pears [6,9,11]. The variations in *STP* gene structure might be due to structural divergence mechanisms, such as the insertion or deletion of exons and introns as well as exonization and pseudoexonization, as suggested by Xu et al. [33]. Interestingly, most of the MeSTPs are basic proteins with p*I* values greater than 7, except for MeSTP14 which has a p*I* value of 6.84. This may be due to the fact that MeSTP14 has a higher proportion of acidic amino acids, including 20 aspartic acids and 22 glutamic acids, which are more than those present in the other MeSTP proteins.

A phylogenetic analysis revealed that the 20 MeSTPs together with the other STP proteins from Arabidopsis and castor bean were classified into four groups, and the MeSTPs grouped with AtSTPs and RcSTPs in each group (Figure 3), suggesting a close relationship between the STPs of these three species and that STP proteins are highly conserved across lineages. Group I contained seven MeSTPs (MeSTP2, 4, 5, 7, 8, 14, and 15), four AtSTPs, and six RcSTPs (Figure 3). Among the AtSTPs belonging to this group, AtSTP6, which exhibited high similarity to MeSTP14, is a high-affinity H^+^/monosaccharide transporter with broad-spectrum substrates, mainly expressed in pollen at the late stage of development [34]. As the homologous gene of *MeSTP7* and *MeSTP*15, *AtSTP*13 has been characterized as a high-affinity hexose-specific/H^+^ transporter expressed in the vascular tissue [14]. Overexpression of *AtSTP*13 increases plant biomass through the regulation of carbon and nitrogen metabolism [35]. *MeSTP*10 and *MeSTP*11 clustered with AtSTP3 in group III (Figure 3). *AtSTP3* is a low-affinity energy-dependent H^+^ symporter detected in green leaves, and the expression of *AtSTP3* is induced by wounding [15]. In group IV, *AtSTP1* is a guard cell-specific localization gene involved in carbon acquisition and plays a possible role in osmoregulation [31]. *AtSTP11* is reported to be a pollen tuber-specific monosaccharide transporter, participating in the allocation of sugars to growing pollen tubes [36]. Therefore, MeSTP proteins clustered with the characterized STP proteins in the same group possibly possess similar biochemical properties. Additionally, a close relationship between the STP members from cassava and castor bean was observed in the phylogenetic tree, reflecting the fact that both cassava and castor bean are members of the *Euphorbiaceae* family, as discussed by Bredeson et al. [37].

In cassava, all of the 20 *MeSTP* genes exhibited differential localization on 18 chromosomes, with some homologous gene pairs (Figure 3 and Figure 4). Among them, three gene pairs (*MeSTP*2*–MeSTP*4, *MeSTP*6*–MeSTP*18, and *MeSTP*17*–MeSTP*19) were determined to be a product of WGD/segmental duplication, whereas two pairs (*MeSTP*4*–MeSTP*5 and *MeSTP*10*–MeSTP*11) were possibly involved in tandem duplication (Figure 4), which is similar to previously reported results in pear according to which six *PbSTPs* could be assigned to WGD/segmental duplication, while five *PbSTPs* were regarded as tandem duplication events [11]. Gene duplication is considered to be the primary driving force for evolution, leading to functional speciation and diversification [38,39]. In this study, the result of gene duplication analysis indicated that the *MeSTP* family has increased rapidly during the course of evolution and that WGD/segmental events have largely contributed to the expansion, accompanied by tandem duplications.

The *STP* genes function in the distribution of monosaccharides, which is further involved in diverse metabolic processes during plant growth and development [40]. Cassava RNA-seq data from public databases were further explored to dissect the expression profiles of the *MeSTP* genes in this study. The results showed that *MeSTP* genes exhibited differential expressions in various cassava tissues (Figure 5A). Similarly, *STP* genes in Arabidopsis also display different expression patterns in various tissues. For example, *AtSTP*1 shows specific expression in the roots and is the major transporter for the uptake of external hexose [12]. *AtSTP*3 exhibits leaf-specific expression and may participate in the retrieval of sugars lost from the cytoplasm by passive leakage [15]. In this study, some of the *MeSTP* genes exhibited tissue-specific expression, such as *MeSTP*2 and *MeSTP*20, which were mainly expressed in the fibrous root, while *MeSTP*5 was specifically expressed in the leaf (Figure 5A). Furthermore, among the 20 *MeSTP* genes, *MeSTP*2 showed the highest expression in the sink tissues (e.g., fibrous root and storage root), and *MeSTP*17 displayed the highest expression in leaf, midvein, petiole, and stem (Figure 5A). In tomato, among different *LeHT* genes, *LeHT*2 shows the highest expression in source leaf, suggesting a possible role of this gene in retrieving cell wall hydrolysis products [41]. The tissue-specific expression analysis of the *MeSTP* genes provides evidence of the potential functions of these gene in specific tissues.

As monosaccharides (e.g., glucose, fructose, and mannose) are important to generate energy and synthesize cellulose and starch, which are further accumulated in the roots of cassava [40], it is important to investigate the expression profiles of *MeSTP* genes in the roots of cassava and dissect the potential roles of these genes in sugar distribution. The expression profiles from the RNA-seq data revealed that most of the *MeSTP* genes are mainly expressed in the roots at early growth stages (Figure 5B). As the expression of *STP* genes is always coupled with that of cell wall invertase genes [2], it can be speculated that sucrose is hydrolyzed in the apoplast by cell wall invertases into monosaccharides and is further transported to the roots by *MeSTPs* at the early root growth stage. However, with the roots developing, a conversion of the sucrose unloading strategy occurs from the apoplastic pathway to the symplastic pathway, which is based on plasmodesmatas and does not employ sugar transporters. This kind of switch was also observed during tuberization in potato and thought to be essential for rapid starch accumulation in developing roots [42]. To study the expression of *MeSTPs* genes in more detail at the early storage root stage, a qRT-PCR analysis was executed. The result revealed that most of the *MeSTPs* displayed higher expression in roots at 30 and 40 days of growth, followed by decreased expression in roots at later growth stages (Figure 6), which further confirmed the in silico data. As the root is a typical sink organ, its development depends on the photoassimilates supplemented from the phloem. The highly expressed *MeSTPs* may function primarily in post-phloem carbohydrate unloading, regulating energy availability for roots growth. Similarly, it has been demonstrated that *AtSTP1* and *AtSTP13* exhibit higher expression in the roots of Arabidopsis, with the possible function of reabsorbing monosaccharides [43]. Furthermore, *AtSWEET4* is characterized as a plasma membrane localization transporter mainly expressed in the stele of roots [44]. In *Medicago truncatula*, *Mest1* (monosaccharide transporter) shows a root tissue-specific expressing pattern to satisfy the high requirement for hexoses in elongating cells [45]. Therefore, *MeSTP* genes exhibiting higher expression in the roots at the early growth stage may be involved in root growth.

In this study, we failed to detect the expression of *MeSTP*1, 6, 9, and *MeSTP*18 in different tissues, development periods, or after stress treatments, thus we could not clone these four full-length genes. It is possible that these four *MeSTPs* are pseudo-genes encoding non-functional sugar transporters. The monosaccharide transport activity of other 16 MeSTPs proteins was further analyzed in a yeast mutant defective in monosaccharide uptake. Most of MeSTPs displayed variations in monosaccharide transport activity despite their similar plasma membrane localization (Figure 7 and Figure 8). Among these MeSTPs, six MeSTP members (MeSTP2, 5, 8, 11, 15, and 19) were able to complement the phenotype of the yeast mutant in the SD medium supplemented with the five tested monosaccharides (Figure 7), suggesting that these MeSTPs transport a broad spectrum of monosaccharide substrates, contributing to general sugar distribution in the roots of cassava. Furthermore, three MeSTP members (MeSTP7, 12, and 17) showed a strong ability to transport mannose, galactose, glucose, and fructose, but not xylose, and MeSTP3 and MeSTP16 exhibited galactose-specific transport activity (Figure 7). The monosaccharides uptake by MeSTPs might be subsequently involved in complex metabolic activities, cell wall reconstruction, or starch synthesis during cassava tuber root growth. For example, it has been reported that Mest1 in *Medicago* is involved in glucose and fructose transportation, facilitating the acquisition of hexoses in root elongating cells [45]. Furthermore, overexpression of AtSWEET4, a plasma membrane localization transporter, in Arabidopsis leads to increasing root growth through the regulation of glucose and fructose uptake from the extracellular space [44]. Therefore, these results suggest that MeSTPs might be vital to monosaccharides distribution, regulating the early tuber root growth of cassava, a hypothesis that merits further study.

## 4. Materials and Methods

### 4.1. Plant Materials

Cassava cultivar “Ku50” was used as plant material in this study. All the cutting seed-stems were normally planted in the field, and the roots were harvested at 30, 40, 45, and 51 days after planting, when the tuber started to form. Furthermore, different cassava tissues, such as leaves, stems, roots, flowers, and seeds were also collected for the preparation of the mixed cDNA template, which was further used for *MeSTPs* cloning. After freezing in liquid nitrogen, all fresh materials were preserved at −80 °C for further total RNA isolation.

### 4.2. Identification of the STP Genes in Cassava

To identify *STP* genes in cassava, the whole-genome data of cassava (*Manihot esculenta v6.1*) were downloaded from the Phytozome website (https://phytozome.jgi.doe.gov/pz/portal.html). The HMMER profile of the Sugar_tr domain (PF00083) from Pfam (http://pfam.sanger.ac.uk/) was used to search the cassava protein database at a standard E-value < 1 × 10^−5^, according to Finn et al. [46]. A total of 24 putative *STP* proteins were identified. Furthermore, the protein sequences of 14 Arabidopsis AtSTPs and 29 rice OsSTPs were used in a BLAST search against the 24 putative *STP* proteins to find their best match sequences. Finaly, a total of 20 STP protein sequences were identified in the cassava genome. The *STP* genes were named *MeSTP*1 to *MeSTP*20 according to their positions on cassava chromosomes.

### 4.3. Gene Structure, Conserved Motifs, and Phylogenetic Analysis

The Gene Structure Display Server (http://gsds.cbi.pku.edu.cn/) was used for the *MeSTP* gene structure analysis. MeSTP protein conserved motifs were analyzed by the MEME and Pfam programs according to the published method of Bailey et al. [47]. MEME was employed using the following parameters: optimum width, 20–60; number of repetitions, any; maximum number of motifs, 12. A phylogenetic tree was constructed based on protein sequence alignment of the 20 MeSTPs, 20 castor bean RcSTPs, and 14 Arabidopsis AtSTPs, through PHYML program using the maximum likelihood (ML) method with 1000 bootstrap replicates, and GTR + I + G was selected as the testing model. Furthermore, neighbor-joining (NJ) phylogenetic trees of the *MeSTPs* gene and protein sequences were constructed using MEGA7 software with 1000 bootstrap resamplings.

### 4.4. Gene Duplication Analysis

The MCScanX program was used to identify *MeSTP* duplications as previously described by Wang et al. [48]. In brief, 33,033 protein sequences from cassava were analyzed using an all-vs-all BLAST search with e-value < 1 × 10^−5^, and the output file format was set to m8. With the BLAST outputs, gene positions file, and the execution of MCScanX program, all genes were classified into various types of duplications. A schematic diagram of the putative duplications of the *MeSTP* genes was constructed using the Circos software [49], and then the putative WGDs/segmental duplications of *MeSTPs* were connected by links.

### 4.5. Expression Profiles of MeSTP Genes in RNA-Seq Data

To explore the expression profiles of *MeSTPs* in different cassava tissues, the fragments-per-kilobase-per-million fragments mapped (FPKM) values of the *MeSTP* genes were obtained from the genome-wide RNA-seq dataset in the Gene Expression Omnibus (GEO) (accession number: GSE82279) submitted by Wilson et al. [30]. Hierarchical clustering was performed using the R package “pheatmap” with the data normalization method of Z-score standardization. The 11 selected cassava tissues were as follows: friable embryogenic callus (FEC), somatic organized embryogenic structures (OES), root apical meristem (RAM), storage root (SR), fibrous root (FR), stem, shoot apical meristem (SAM), lateral bud (LB), midvein (MV), petiole, and leaf (LF).

To explore the expression profiles of *MeSTPs* in roots at different growth stages, including the early storage root (75 days), the medium tuber root (120 days), and the late storage root (150 days), the public high-throughput RNA-seq read archive (SRA) databases of the cassava cultivar “KU50” submitted by the Chinese Academy of Tropical Agricultural Sciences [21] were used. The FASTX-toolkit program (version 0.0.13, http://hannonlab.cshl.edu/fastx_toolkit/) was used to remove the adapter sequence from the raw sequence reads. The obtained clean reads were aligned to the cassava reference genome (version 6.1) derived from the Phytozome website, using Tophat 2.0.11 with default parameters [50]. The FPKM values were calculated by Cufflinks as described by Trapnell et al. [51]. Hierarchical clustering was performed as described above.

### 4.6. Quantitative RT-PCR (qRT-PCR) Analysis of MeSTP Genes

Total RNA from the roots of the cassava cultivar “KU50” was extracted using RNAplant Plus reagent (Tiangen, China) according to the manual. First-strand cDNA was synthesized from 2 µg of DNase-treated RNA using M-MLV reversed transcriptase (Promega, Madison, WI, USA). SYBR Premix Ex Taq II (Takara, Japan) was used for the qRT-PCR analysis, which was monitored on an Applied Biosystems 7500 Real-Time PCR system (Thermo, Waltham, MA, USA). The specific primer pairs of 20 *MeSTP* genes used for qRT-PCR analysis are shown in Supplementary Table S1. The relative expression levels were calculated by the 2^−ΔΔ*C*t^ method [52]. Each gene expression analysis had three biological replicates.

### 4.7. Complementation Analysis of MeSTPs in Yeast (Saccharomyces cerevisiae)

The open reading frames (ORFs) of *MeSTP* genes were amplified from cassava cDNA libraries constructed by Phusion High-Fidelity DNA Polymerase (Thermo, USA) using gene-specific primers (Supplementary Table S2). The amplified products were gel-purified and cloned into the yeast shuttle vector pDR195 using the Clontech In-Fusion PCR Cloning Kit (Takara, Japan). Vector pDR195 contains the *URA3* as a selective marker and the *Pma1* promoter for constitutive expression of the target gene.

The yeast strain EBY. VW4000, which is deficient in hexose transport as a result of the multiple knock-out of endogenous transporters [53], was used to analyze the functions of *MeSTP* genes. The plasmid constructs were transferred into EBY.VW4000 as described by Morita et al. [54]. Yeast cells transformed with the empty vector pDR195 was used as a control. The transformed cells were first precultured in liquid synthetic deficient (SD)-ura medium supplemented with 2% maltose as the sole carbon source until the OD_600_ value reached 0.6. Three serial dilutions (10×) of yeast cells were dropped on solid SD-ura medium containing 2% maltose or different monosaccharide substrates (including mannose, galactose, xylose, glucose, and fructose) and cultured for 3 days at 27 °C.

### 4.8. Subcellular Localization of MeSTP

The ORFs of *MeSTP* genes were amplified using gene-specific primers (Supplementary Table S3) and then cloned into pX-DG vector fused with GFP (green fluorescent protein) according to Chen et al. [55]. The plasmid constructs were then transiently expressed in cassava mesophyll protoplasts as previously described by Wu et al. [56]. The GFP fluorescence was imaged using a confocal laser scanning microscope (Leica TCS SPII, Leica Microsystems, Wetzlar, Germany).

## 5. Conclusions

In this study, a total of 20 *MeSTP* genes were identified in a genome-wide survey of the cassava genome. All *MeSTPs* were classified into four groups and contained the Sugar_tr conserved motifs. Gene duplication analysis showed that WGD/segmental and tendem duplications were involved in the expansion of the *MeSTP* genes. The RNA sequencing results of 11 tissues of cassava indicated that *MeSTPs* is essential for the development of mutiple tissues in cassava. Furthermore, comparisons of the expression levels of the *MeSTP* genes during storage root development showed that *MeSTPs* are mainly expressed at the early storage root, indicating that there may be a switch in sucrose unloading pathways during the maturation of the storage root. Our results of qRT-PCR, monosaccharide transport activity, and subcellular localization of MeSTPs, confirm that MeSTPs are plasma membrane localization transporters critical to monosaccharides distribution, which regulate early tuber root growth in cassava.

## Figures and Tables

**Table 1:** Information about the *STP* genes in cassava.

Gene Name	Gene Locus	CDS Length (bp)	AA ^a^	MW ^b^ (kDa)	p*I* ^c^	TMD ^d^
*MeSTP*1	Manes.01G067100	1560	519	57.08	8.51	11
*MeSTP*2	Manes.01G164600	1566	521	57.48	10.06	12
*MeSTP*3	Manes.01G182200	1392	463	51.28	9.4	10
*MeSTP*4	Manes.02G122000	1575	524	57.37	9.91	12
*MeSTP*5	Manes.02G122100	1536	511	56.51	9.08	11
*MeSTP*6	Manes.03G025000	1530	509	55.91	8.81	10
*MeSTP*7	Manes.03G180400	1590	529	58.25	9.26	12
*MeSTP*8	Manes.03G180600	1482	493	54.36	9.02	11
*MeSTP*9	Manes.04G053100	1545	514	56.31	7.44	12
*MeSTP*10	Manes.06G132600	1524	507	55.70	9.34	10
*MeSTP*11	Manes.06G132700	1527	508	55.37	9.61	10
*MeSTP*12	Manes.11G102900	1545	514	56.07	8.85	12
*MeSTP*13	Manes.14G139800	1548	515	55.51	9.08	11
*MeSTP*14	Manes.15G027100	1548	515	56.84	6.84	12
*MeSTP*15	Manes.15G027300	1596	531	58.54	8.85	12
*MeSTP*16	Manes.15G030700	1548	515	56.21	8.97	12
*MeSTP*17	Manes.16G020100	1575	524	57.85	9.16	12
*MeSTP*18	Manes.16G111500	1329	442	48.97	9.94	8
*MeSTP*19	Manes.17G033300	1569	522	57.58	8.9	12
*MeSTP*20	Manes.18G067900	1347	448	48.74	10.09	11

^a^ Length of the amino acid sequence; ^b^ Molecular weight of the amino acid sequence; ^c^ Isoelectric point of the MeSTP; ^d^ Number of transmembrane domains, as predicted by the TMHMM Server v2.0.

**Table 2:** Twelve different motifs commonly observed in cassava *STP* proteins.

Motif	Length	Protein Sequences	Pfam Domain
1	80	WSWGPLGWLVPSEIFPLETRSAGQSITVCVNMLFTFVIAQCFLTMLCHMKYGIFLFFAGWIVIMTIFVYFLLPETKNIPI	Sugar_tr
2	82	NNYCKYDNQYLQLFTSSLYLAALVASFFASYVTRKYGRKPSMQVGSISFCAGAILNAAAQNVWMLIIGRCLLGCGVGFANQA	Sugar_tr
3	41	CMIAAMGGLMFGYDIGISGGVTSMDDFLKKFFPTVYRKKHH	Sugar_tr
4	35	IPFFQQLTGINVIMFYAPVLFQTMGFGDDASLYSA	Sugar_tr
5	41	VPLYLSEMAPPKYRGALNICFQLTITIGILIANLINYGTEK	Sugar_tr
6	41	HPWGWRLSLGLAAVPALIMTVGSLFLPETPNSLIERGHHEE	Sugar_tr
7	49	VITGAVNVISTLVSMYTVDKWGRRVLFLEAGIQMFICQVAVGCCLAAHF	Sugar_tr
8	21	AKQVKHPWRNLMKRKYRPQLV	
9	21	EEMDRVWKNHWFWKRYMDDDD	
10	21	LKKIRGTDNVDEEFDDLVDAS	
11	21	LPKGYAIFVVCMICVYVAGFA	
12	15	AHDYEGKITPYVIVC	

## Supplementary Materials

Supplementary materials can be found at http://www.mdpi.com/1422-0067/19/4/987/s1.
